# Glycan–protein cross-linking mass spectrometry reveals sialic acid-mediated protein networks on cell surfaces †

**DOI:** 10.1039/d1sc00814e

**Published:** 2021-05-18

**Authors:** Yixuan Xie, Siyu Chen, Qiongyu Li, Ying Sheng, Michael Russelle Alvarez, Joeriggo Reyes, Gege Xu, Kemal Solakyildirim, Carlito B. Lebrilla

**Affiliations:** Department of Chemistry, University of California Davis California USA; Department of Chemistry, Biochemistry, Molecular, Cellular and Developmental Biology Graduate Group, University of California Davis California USA; Department of Biochemistry, University of California Davis California USA; Institute of Chemistry, University of the Philippines Los Baños Laguna Philippines; Marine Science Institute, University of the Philippines Diliman Quezon City Philippines; Department of Chemistry, Erzincan Binali Yildirim University Erzincan Turkey cblebrilla@ucdavis.edu

## Abstract

A cross-linking method is developed to elucidate glycan-mediated interactions between membrane proteins through sialic acids. The method provides information on previously unknown extensive glycomic interactions on cell membranes. The vast majority of membrane proteins are glycosylated with complicated glycan structures attached to the polypeptide backbone. Glycan–protein interactions are fundamental elements in many cellular events. Although significant advances have been made to identify protein–protein interactions in living cells, only modest advances have been made on glycan–protein interactions. Mechanistic elucidation of glycan–protein interactions has thus far remained elusive. Therefore, we developed a cross-linking mass spectrometry (XL-MS) workflow to directly identify glycan–protein interactions on the cell membrane using liquid chromatography-mass spectrometry (LC-MS). This method involved incorporating azido groups on cell surface glycans through biosynthetic pathways, followed by treatment of cell cultures with a synthesized reagent, *N*-hydroxysuccinimide (NHS)–cyclooctyne, which allowed the cross-linking of the sialic acid azides on glycans with primary amines on polypeptide backbones. The coupled peptide–glycan–peptide pairs after cross-linking were identified using the latest techniques in glycoproteomic and glycomic analyses and bioinformatics software. With this approach, information on the site of glycosylation, the glycoform, the source protein, and the target protein of the cross-linked pair were obtained. Glycoprotein–protein interactions involving unique glycoforms on the PNT2 cell surface were identified using the optimized and validated method. We built the GPX network of the PNT2 cell line and further investigated the biological roles of different glycan structures within protein complexes. Furthermore, we were able to build glycoprotein–protein complex models for previously unexplored interactions. The method will advance our future understanding of the roles of glycans in protein complexes on the cell surface.

## Abstract 2

The cell surface glycocalyx is highly interactive defined by extensive covalent and non-covalent interactions. A method for cross-linking and characterizing glycan–peptide interactions *in situ* is developed.

## Graphical abstract

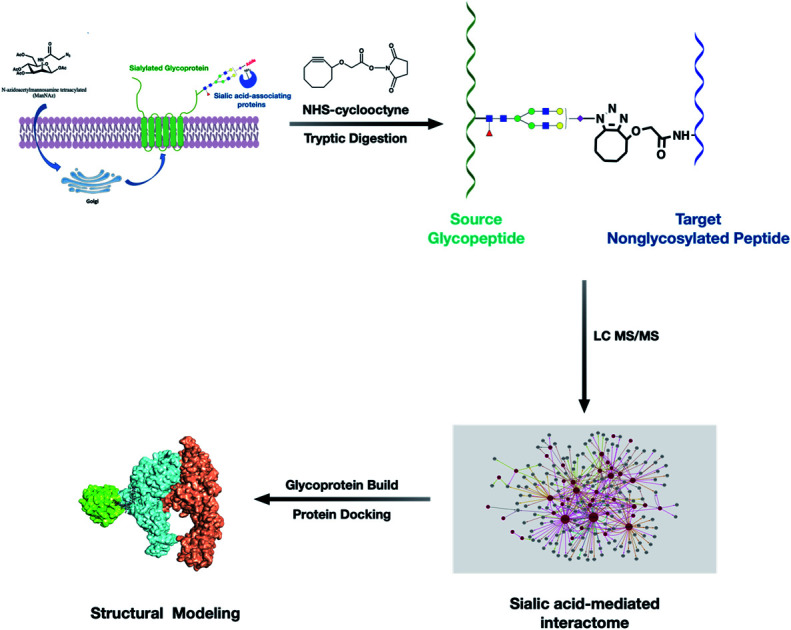

## 1. Introduction

Cell surfaces are covered with a matrix of glycans on a scaffold of proteins and lipids. In particular, sialic acids at the terminus of glycans creates a highly interactive environment that governs many cellular functions such as cell–cell signaling, cell adhesions, and immunological recognition.^1–3^ However, the nature of such weak and transient interactions impedes the systematic identification of the interacting complexes within living cells or tissues using existing analytical methods. In recent years, mass spectrometry (MS)-based methods including proximity labeling, hydrogen–deuterium exchange (HDX), affinity purification combined with mass spectrometry (AP-MS), and cross-linking mass spectrometry (XL-MS) have been extensively developed, which provide effective tools for characterizing protein–protein interactions.^4–6^ Recently, Zhang *et al.* combined HDX with XL-MS, and characterized interleukin-7 and its α-receptors with largely enhanced spatial resolution.^7^ However, these studies have rarely focused on characterizing glycoprotein–protein interactions due to the limitations in glycan analysis, despite the fact that glycans are vital mediators of many intercellular and extracellular interactions.^8^

The inherent problems in the analysis are mostly due to the low abundance of glycoproteins and the large diversity of glycoforms that further complicates the analysis. With the development of proteomic and glycoproteomic techniques, the methods that characterize protein complexes and networks on cell membranes involving glycan–protein interactions have been explored. Paulson, Kohler, and their respective colleagues have developed diazo-bearing sialic acid reporters to capture *cis*- and *trans*-interactions of CD22.^9–11^ They used the metabolic labeling method to incorporate diazo groups into sialic acids. Proteins that associate with sialic acids can then be captured by producing reactive carbenes through ultraviolet irradiation. However, only isolated binding-pairs can be characterized because the method relied on purification by gel electrophoresis. To explore proteome-wide interactions, we have developed the protein oxidation of sialic acid environments (POSE) method to identify potential sialic acid-associating proteins *in situ*.^12^ Frei *et al.* employed a trifunctional chemoproteomic cross-linker and created a prominent ligand-based receptor-capture (LRC) technology to identify glycan-binding proteins.^13^ The targets of vaccinia viruses on the human cell surface have been successfully illustrated by this method. The same group further improved the method termed HATRIC-LRC, which successfully identified the receptors of endothelial growth factor receptor (EGFR) antibody and Holo-transferrin (TRFE) from cells.^14^ However, glycan information within the interactions could not be obtained, and the glycan-mediated protein networks on the cell surface were absent. Moreover, the method involves the use of periodate, which can potentially disrupt the interaction of native glycans and proteins.

**Fig. 1:**
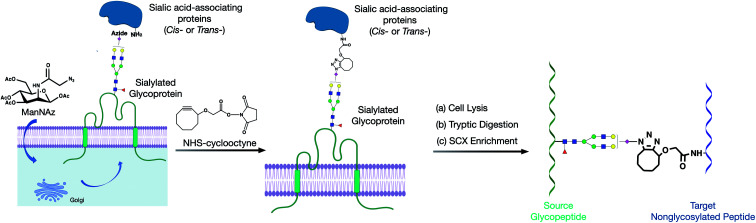
Proposed method for producing and implementing glycan–protein cross-linking on the cell membrane of cell lines. (a) The metabolic incorporation of the azido groups into cell surface glycoproteins as SiaNAz. (b) The cross-linking reaction of NHS–cyclooctyne to the azido groups and primary amines. (c) The cross-linked glycopeptide–peptide product was obtained with subsequent cell lysis, tryptic digestion, and SCX enrichment.

Herein, we developed a cross-linking method to characterize glycan–peptide cross-linked (GPX) products coupled in cross-linking reactions, which provided direct information on the sialic acid-mediated protein networks on cell membranes (Fig. 1). In our method, *N*-azidoacetylmannosamine (ManNAz) was metabolically incorporated into cell surface sialic acid-containing glycoproteins through the *de novo* biosynthetic pathway. This bioorthogonal reporter was further conjugated with the cyclooctyne functional group by the addition of a synthesized *N*-hydroxysuccinimide–cyclooctyne (NHS–cyclooctyne) reagent. An NHS ester-activated carboxylic acid reagent was further added to react with primary amines on the lysine side chains of nearby proteins. After the glycan–protein cross-linking step, the cells were lysed and tryptic digested through a previously reported workflow.^15^ The glycan–peptide cross-linked (GPX) pairs were purified using the reverse-phase column and the strong cation exchange (SCX) cartridge and analyzed by reversed phase liquid chromatography coupled with a high-resolution Orbitrap mass spectrometer. Enriched GPX pairs were fragmented using high energy collision-induced dissociation (HCD) for identification through a modified proteomics workflow. Although the NHS–cyclooctyne cross-linker is not MS-cleavable, the glycans in the GPX pairs can be fragmented during tandem MS/MS, which makes the pairs behave as MS-cleavable cross-linked peptide pairs with different glycan compositions. The complexity derived from glycan heterogeneity daunted the direct identification using the search software. To address this problem, we obtained site-specific glycopeptide information from the specific cell line through glycoproteomic analysis using LC-MS. In this way, glycans containing SiaNAz were mapped with protein site information allowing us to narrow down the glycopeptides, thereby largely avoiding misidentification from searching a much larger combinatorial space of peptides. By integrating the glycomic and glycoproteomic results with available cross-linking software (MeroX), we could unambiguously identify the GPX products.^16^ The characterization of the GPX products coupled in cross-linking reactions provided direct information regarding the sialic acid-mediated protein networks on cell membranes. We further illuminated the glycan–protein networks present on the PNT2 cell membrane which mediate the function of cells.

## 2. Methods

### 2.1 ManNAz treatment in PNT2 cells

Human immortalized normal prostatic epithelial PNT2 cells were obtained from ATCC and grown in Roswell Park Memorial Institute (RPMI) 1640 medium, while supplemented with 10% (v/v) fetal bovine serum (FBS), 100 U ml^−1^ penicillin and 100 μg ml^−1^ streptomycin. The cells were grown at 37 °C in a humidified incubator with 5% CO_2_. At 60% cell confluency, the cells were treated with 100 μM ManNAz in cell culture media and let to stand in the incubator for 72 hours.

### 2.2 Cross-linking of glycan–protein interaction on the cell surface

For the cross-linking reaction, 1 × 10^7^ cells were treated with a 100 μM synthesized cross-linker in PBS for 60 minutes at 37 °C. The reaction was quenched with 0.1 M tris-buffer for 5 minutes at 37 °C and washed with PBS three times. The cells were harvested with scrapers and resuspended in a homogenization buffer containing a protease inhibitor cocktail (EMD Millipore, CA), 0.25 M sucrose, and 20 mM HEPES–KOH (pH 7.4). The cells were then lysed at 4 °C using a probe sonicator (Qsonica, CT) with alternating on and off pulses of 5 and 10 seconds, respectively.

### 2.3 Tryptic digestion of proteins

The lysed cell pellets were reconstituted with 60 μL of 8 M urea and sonicated for 15 minutes for denaturing. 2 μL of 50 mM dithiothreitol (DTT) solution was then added to the samples followed by incubation at 55 °C for 50 minutes. Thereafter, 4 μL of 450 mM iodoacetamide (IAA) was added to alkylate the free thiol groups of the denatured proteins for 25 minutes at room temperature in the dark. After diluting the urea concentration and adjusting the pH of the samples by adding 420 μL of 50 mM ammonium bicarbonate (NH_4_HCO_3_) solution, 3 μg of trypsin was added to the mixture, and protein digestion was conducted at 37 °C for 18 hours. The resulting peptides were desalted by solid-phase extraction with C18 cartridges containing 500 mg of materials. The cross-linked samples were further enriched using SCX cartridges based on previous procedures. The samples were dried *in vacuo* using miVac (SP Scientific, PA).

### 2.4 Analysis of glycan–protein cross-linked (GPX) products

The GPX samples were reconstituted in H_2_O and characterized using the UltiMateTM WPS-3000RS nanoLC system coupled with an Orbitrap Fusion Lumos (Thermo Fisher Scientific). A 1 μL solution of each sample was injected, and the analytes were separated on an AcclaimTM PepMapTM 100 C18 LC Column (3 μm, 0.075 mm × 500 mm, Thermo Fisher Scientific) at a flow rate of 300 nL min^−1^. Water containing 0.1% formic acid and 80% acetonitrile, and water containing 0.1% formic acid were used as solvents A and B, respectively. The LC gradient was 0–5 min, 4–4% (B); 5–130 min, 4–35% (B); 130–150 min, 35–50% (B); 150–153 min, 50–100% (B); 153–168 min, 100–100% (B); 168–170 min, 100–4% (B); and 170–180 min, 4–4% (B). MS spectra were collected within a mass range of *m*/*z* 300–1800 in the positive ionization mode. The filtered top ten precursor ions were subjected to fragmentation through 30 ± 3% higher-energy C-trap dissociation (HCD) with nitrogen gas, and the precursor ions with the same *m*/*z* were excluded within the following 45 seconds.

### 2.5 Search of GPX pairs using the MeroX software

The raw spectra were converted to .mgf files and searched with the MeroX software (v.2.0.1.4) using a modified human protein FASTA database acquired from UniProt. The database generated from the glycoproteomic results was used to assign *N*-glycosites. The identified *N*-glycosites were annotated as glycosylated asparagine (“J” amino acid), which has the same composition as the nonglycosylated asparagine. The number of missed cleavages was restricted to 2. The precursor mass tolerance was limited to 20 ppm, and the fragmentation mass tolerance was limited to 0.1 Da. Carbamidomethylation at cysteine was assigned as the fixed modification, and the oxidation at methionine was selected as the variable modification. The common mass offsets of the glycopeptide, such as 203.08 Da and 406.16 Da corresponding to the single- and double-HexNAc-modified peptides, were added as the information of fragment modifications. Our initial analysis used the quadratic mode instead of the RISE and RISEUP modes because the required peptide + cross-linker fragments of the two peptides were either lacking or highly variable. The identified cross-linked peptides with high confidence were filtered through a minimum peptide score of 80. The overall score was not evaluated as the scoring system of MeroX was based on PPX and was not suitable for GPX products.

### 2.6 Glycomic and glycoproteomic analyses of cell surface glycoproteins

The detailed procedures were reported previously.^15^ Briefly, the *N*-glycans were released from cell membrane using PNGase F after overnight incubation at 37 °C. The released *N*-glycans were purified using porous graphitic carbon (PGC) SPE plates, the glycan samples were analyzed with an Agilent 6520 Accurate Mass Q-TOF LC/MS equipped with a PGC nano-chip (Agilent, CA), and the results were extracted with the MassHunter Qualitative Analysis B08 software (Agilent, CA). For glycoproteomic analysis, glycopeptides after trypsin digestion were enriched by solid-phase extraction using iSPE®-HILIC cartridges (Nest Group, MA). The enriched glycopeptides were characterized using a UltiMate™ WPS-3000RS nanoLC system coupled with an Orbitrap Fusion Lumos (ThermoFisher Scientific), and an in-house human *N*-glycan database was applied to the raw results using the Byonic software (Protein Metrics, CA).

### 2.7 Protein docking

From our cross-linking experiment, the Hex_(5)_HexNAc_(4)_Fuc_(1)_Sia_(1)_ and Hex_(5)_HexNAc_(4)_Fuc_(1)_Sia_(2)_ glycans on ITGB1 Asn97 were found to cross-link with RAP1B Lys104. To corroborate the cross-linking results, modeling studies were conducted. Specifically, DisVis was used to calculate distance restraints in cross-linked proteins for specific glycan sizes (10–30 Å). Putative protein complexes were then generated using the HADDOCK software. Out of 66 structures, the complex with the highest HADDOCK score was subsequently glycosylated using the CHARMM-GUI glycan modeler.

## 3. Results

### 3.1 Method validation

The crosslinker was synthesized as described in the ESI.† It was designed to take advantage of bioorthogonal methods that produce activated glycans on the cell membrane so that click chemistry can be applied on one end and a reaction with free amines on the other. To evaluate first the reactivity of the click chemistry *in situ*, cells were fed ManNAz to produce SiaNAz on the cell membrane. We then treated cells with just the cyclooctyne carboxylic acid (no NHS terminus) to act as a mock linker. The *N*-glycans released from the cell membrane after the click reaction of the mock linker were monitored using tandem MS to observe the extent of reaction and to obtain structural information on the reacted glycan (Fig. S1†). These experiments were performed repeatedly and the sialylated glycans (with SiaNAz) were reacted toward 70% completion in the 1 hour reaction period. The NHS ester-amine reaction was characterized in an earlier study, and the reaction was found to reach nearly complete (>95%) within 1 hour at 37 °C.^17^ The copper-free click and NHS ester–amine trials were useful as they indicated that they were biocompatible under physiological conditions and both exhibited high efficiencies and specificities within the same reaction period.

**Fig. 2:**
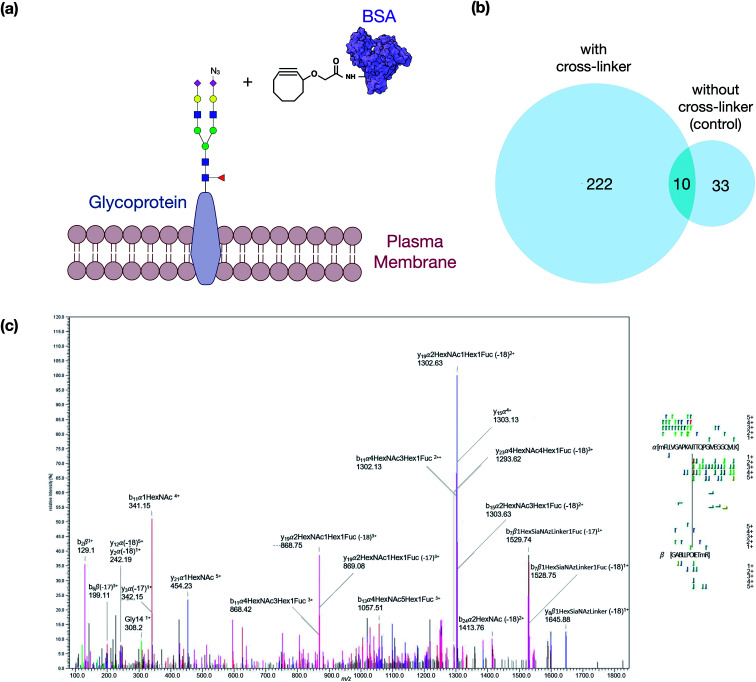
Overview of the method optimization. (a) The schematic diagram represents the modified BSA cross-linking concept. (b) Comparison of the identified GPX pairs from the experiment with cross-linker treatment and control (without treatment). (c) Cross-linked peptides generate fragmentations in HCD, as shown in the spectrum of an inter-linked peptide MFLLVGAPKAN(74)TTQPGIVEGGQ-Hex_(5)_HexNAc_(4)_Fuc_(1)_Sia_(1)_-GACLLPK(204)IETMR.

We then optimized the workflow with experiments using a selected target protein. In these experiments, bovine serum albumin (BSA) was modified with the cross-linker to yield proteins with pendant cyclooctyne (Fig. 2a). The modified protein was then introduced to the cell culture to allow the cyclooctyne to react with SiaNAz-containing glycans on the cell membrane. Tryptic digestion was then performed on the cell lysates to yield the GPX products containing a glycopeptide from a cell membrane glycoprotein and a BSA peptide. The resulting pairs were analyzed by LC-MS, and the generated data were used to evaluate various cross-linking software that are currently available.

The best suited informatic tools for identifying peptide–peptide cross-links (PPX) with LC-MS included pLink, XlinkX, and MeroX.^16,18,19^ Based on our evaluation, MeroX was found the most effective for identifying GPX products. Using MeroX, the false-discovery rate (FDR) was decreased to less than 5% while filtering out peptide scores less than 80 (Fig. 2b). To confirm the reproducibility of the method, BSA-cross-linked samples were analyzed in triplicates with more than 60% overlap of identified GPX pairs obtained (not shown).

We also optimized the collision conditions in the higher-energy C-trap dissociation (HCD) for fragmenting GPX pairs. The HCD method was then sufficient for fragmenting GPX pairs and yielded high quality information for confident identification. For example, the MS/MS spectrum of a GPX product shown in Fig. 2c readily identified the glycopeptides as the membrane glycoprotein ITGAV (integrin alpha V) cross-linked to a peptide of BSA. Other representative spectra are shown in Fig. S2.† The glycopeptide–peptide cross-link did not behave like the more common peptide–peptide cross-link. The former, due to its significantly higher mass, required more energy to fragment. Controlling the HCD energy without strong attenuation of the signal proved difficult. Therefore, the tandem MS appeared over-fragmented compared to the peptide–peptide cross-link spectrum in some cases, but were nonetheless sufficient to identify the sequences. It was noted that the presence of the cross-linking reaction on lysine or arginine resulted in a missed cleavage site. We also examined other factors including the incorporation of ManNAz, the efficacy of cross-linking reaction on cell lines, and the enrichment of GPX products to validate the method further. Details of these experiments are provided in the ESI.†

### 3.2 Cross-linking on the cell membrane

Cross-linking reactions were performed *in vivo* using PNT2 cells. This cell line was selected because the surface glycome is highly sialylated and represented normal prostate epithelium cells. More than 300 unique glycoprotein–protein pairs were found in the chromatogram (Fig. S3†). The number of unique glycopeptide–peptide combinations was far greater with numbers corresponding to approximately 500. We observed that the number of identified GPX pairs was associated with the relative abundance of each glycoform. The bi-antennary monosialylated monofucosylated glycan, Hex_(5)_HexNAc_(4)_Fuc_(1)_Sia_(1)_, was the most abundant glycan on the PNT2 cell membrane, and indeed many of the identified GPX pairs were cross-linked through this glycan. The less abundant glycan Hex_(5)_HexNAc_(4)_Sia_(1)_ only produced limited interactions. Based on the data, over 40 glycoproteins were identified as the source proteins in the GPX experiments. This represents a large fraction of the over 70 sialylated glycoproteins annotated in the proteomic analysis using the UniProt database.

The target proteins were presumed to be some type of sialic acid-binding proteins. Indeed, functional analysis of the target proteins yielded binding proteins, with both anionic and cationic binding functions. The proteins with anion binding functions were consistent with glycan-mediated interactions and the anionic nature of sialic acids, while the proteins with cation binding functions were mostly associated with an alkali or alkaline earth metal and act like typical C-type lectins. Comparison of the target proteins to sialic acid-binding proteins identified earlier through oxidative proximity labeling experiments yielded a nearly 50% overlap in the total proteins identified (Fig. S4†).^12^ Additionally, a greater than 70% overlap was observed when comparing the protein complexes identified here and in the interaction that was previously identified, which confirmed the consistency of the results and suggested sialic acids may mediate these interactions. Meanwhile, the other 30% of the proteins not found as sialic acid binding proteins in published databases should be assigned as potential glycan-binding proteins (Fig. S5†).

**Fig. 3:**
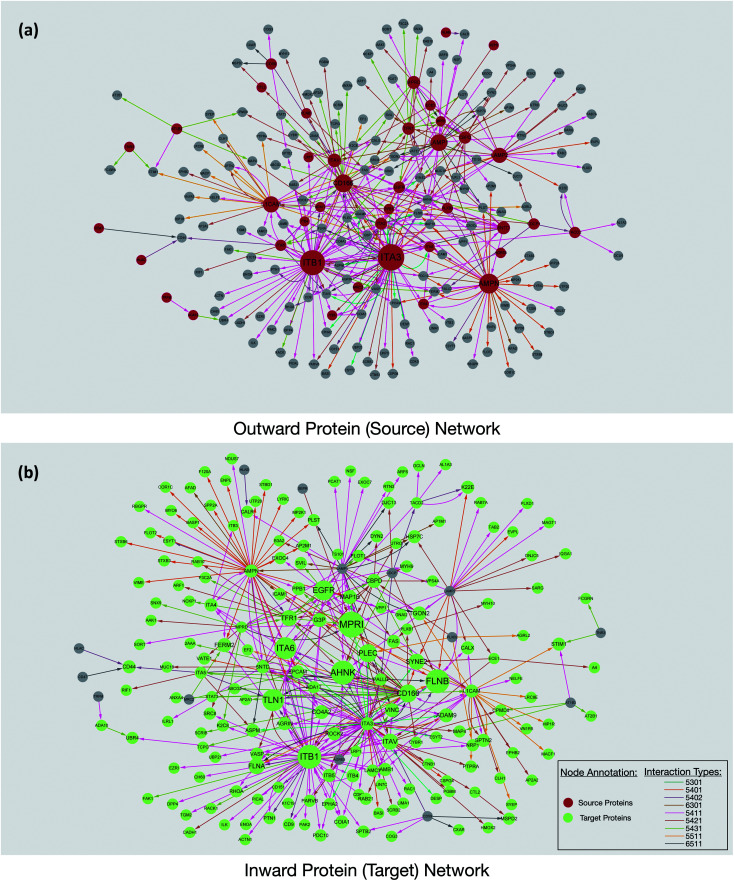
The protein interaction networks revealed from the PNT2 cell line. (a) The network was constructed based on the glycoproteins (red) interacting with other proteins through their glycans. The size of the circle is proportional to the number of proteins involved in the interaction. (b) A network corresponding to a protein that interacted with glycoproteins through other protein glycans. The glycan linkage was annotated based on Hex_(*n*)_HexNAc_(*n*)_Fuc_(*n*)_Sia_(*n*)_ and colored as in the legend.

Interaction maps were constructed using Cytoscape and visualized by grouping the source glycoproteins (Fig. 3a) and their protein targets (Fig. 3b). From the interaction maps, we noted the emergence of hub proteins that interacted through glycans with several other proteins simultaneously. The hubs were either glycoproteins that radiated their glycans toward many other proteins (outward hubs), or proteins that interacted with many glycoproteins through the other proteins' glycosylation (inward hub). The presence of these hubs suggested that key proteins control or are controlled by other proteins through the sialic acid-mediated interaction network.

Outward hubs were glycoproteins associated with many target proteins through their sialylated glycans. Sialic acids likely play a role in the function of these glycoproteins. Integrins, especially ITB1 (integrin beta-1) and ITA3 (integrin alpha-3), were noted to be large outward hubs. Integrins are known to participate in many biological functions including binding, signaling, and cell adhesions.^20^ Other sialylated glycoproteins including AMPN (aminopeptidase N), CD166, L1CAM (neural cell adhesion molecule L1), LAMP1, and LAMP2 (lysosome-associated membrane glycoprotein 1 and 2) were also noted to be prominent outward hubs. The biological functions of source proteins and target proteins were further analyzed using the STRING software. As shown in Fig. S6,† nearly 50% of the source proteins were involved in cell adhesion, while only 12% of the target proteins were found with similar functions, suggesting the unique role of sialic acid in cell adhesion and migration. This result demonstrated hub glycoproteins likely play major roles in cellular attachments. Indeed, the sialylated CD166 protein, a dominant outward hub, is known to be involved in inhibiting monocyte cell migration.^21^ Additionally, the two LAMP proteins were reported to mediate cell–cell adhesion through sialic acids.^22,23^

The non-glycosylated protein AHNAK (also known as desmoyokin) was found to be an inward hub and a common target of many glycoproteins. This very large protein has been shown to be important with a multitude of roles including as a structural and signaling protein on cell membranes.^24^ It was previously reported that the downregulation of AHNAK was accompanied with high tumor potential.^25^ Proteins including FLNB (filamin-B), and TLN1 (talin-1) were also found to be inward hubs. FLNB and TLN1 were reported to interact with integrins, and the inhibition of sialylation was reported to decrease their association.^26^

Interestingly, glycosylated proteins were also found as inward hubs. For example, EGFR was a dominant outward connected protein such as integrins; however, it was also the glycan target of several integrins, suggesting that the interactions between these proteins are extensively mediated by glycans. We compared all the source proteins and target proteins with the lipid raft database,^27^ and over 80% and 60% of the source and target proteins can be found from the lipid rafts, respectively (not shown). This result is consistent with the previous observation from confocal microscopy and supports the evidence that glycoproteins are often associated with the lipid raft microdomains on the cell membrane.^12^ Overall, our results from the network integration analysis demonstrated the emergence of hub proteins that appear to play a central role in the sialic acid-mediated interactome.

### 3.3 Glycan structures can modulate protein–protein interactions

With the glycan–protein interaction networks mapped, we further investigated fundamental questions regarding the role of specific glycan types in their selection of protein targets. We first examined the role of fucosylation by noting the protein target with and without fucosylation of the glycan. The first fucose on *N*-glycan is generally located at the base of the chitobiose core, thus mono-fucosylated *N*-glycan would most likely be core fucosylated.^28^ The comparison of a non-fucosylated and a core fucosylated glycan with regard to their target proteins should provide indications of the role of core fucosylation. We used pairs with similar glycan compositions that differed only in fucosylation. For example, Hex_(5)_HexNAc_(4)_Fuc_(1)_Sia_(1)_ had the same glycan composition as Hex_(5)_HexNAc_(4)_Sia_(1)_ with the exception of a single fucose. The two structures had abundances of the same degree of magnitude, with Hex_(5)_HexNAc_(4)_Sia_(1)_ having 45% of the abundance of Hex_(5)_HexNAc_(4)_Fuc_(1)_Sia_(1)._ However, the number of proteins that interacted with Hex_(5)_HexNAc_(4)_Fuc_(1)_Sia_(1)_ was nearly ten times greater than that for Hex_(5)_HexNAc_(4)_Sia_(1)_. Further investigation of the protein interactions of the two glycan compositions showed that the number of intra- (within the same protein) *versus* inter- (different protein) protein interactions differed for the two compositions (Fig. S7†). The structure with core fucosylation (Hex_(5)_HexNAc_(4)_Fuc_(1)_Sia_(1)_) primarily interacted with other proteins, with inter-protein interactions representing nearly 100% of the GPX product compared to 80% of the intra-protein product. However, the non-core fucosylated structures (Hex_(5)_HexNAc_(4)_Sia_(1)_) produced as much as 25% intra-protein interactions compared to nearly zero for the core fucosylated structures. Therefore, it appeared that core fucosylation is likely used to enhance inter-protein interactions, while the non-fucosylated structures can enhance intra-protein interactions.

We also investigated the effect of the degree of sialylation and fucosylation on protein association using the same base glycan structures with different amounts of sialylation and fucosylation. For example, Hex_(5)_HexNAc_(4)_Sia_(1)_ is a biantennary monosialylated structure, while Hex_(5)_HexNAc_(4)_Sia_(2)_ is the same base glycan composition with an additional sialic acid. For the two glycans, only a small fraction (4 out of 22) of their target proteins overlap, specifically ITA2 (integrin alpha-2), FLNB (filamin-B), PALLD (palladin), and K22E (keratin, type II cytoskeletal 2 epidermal). However, the same glycans when observed over replicates generally yielded over 60% similarities.

We further investigated the potential biological significance of increasing (or decreasing) the extent of sialylation. For example, 65% (11 out of 17) of the proteins that interacted with di-sialylated glycan (Hex_(5)_HexNAc_(4)_Sia_(2)_) having cell surface receptor signaling function, while only 30% (10 out of 29) of mono-sialylated glycan (Hex_(5)_HexNAc_(4)_Sia_(1)_) targeted proteins with similar functions. This result suggested that the change of glycoprotein targets might be the requirement for some cellular processes. Indeed, the activity of some cell signaling proteins was previously found depending on the degree of cell surface sialic acid.^29,30^ Similarly, we examined the role of multiple fucosylation on the selection of target proteins by examining glycan compositions with the same base structures and varying amounts of fucosylation. The first fucose is generally on the core (with exceptions), while the second and third are in the antennae. The effect of multiple fucosylation was more difficult to establish; however, we noted that glycans with only core fucose and glycans with antennary fucose had different preferences for selecting targets. The proteins CTNA2 and CTNB1 were previously found to interact with α(1–2)-linked antennary fucose specifically.^31^ Indeed, CTNA2 (catenin alpha-2) and CTNB1 (catenin beta-1) were only captured by glycans with antennary fucose (Hex_(5)_HexNAc_(4)_Fuc_(2)_Sia_(1)_), but not by glycans with only core fucose (Hex_(5)_HexNAc_(4)_Fuc_(1)_Sia_(1)_).

**Fig. 4:**
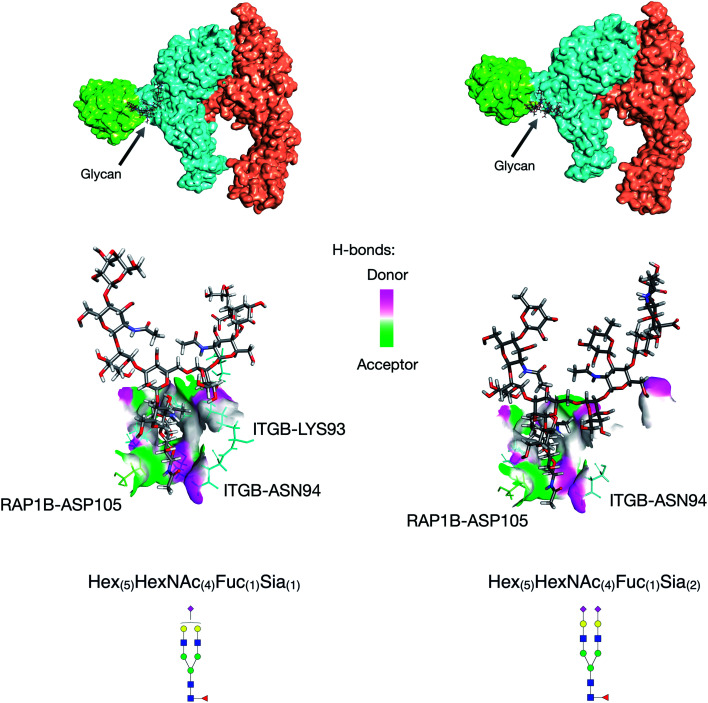
Protein docking results for the ITGB1-RAP1B interaction. Hex_(5)_HexNAc_(4)_Fuc_(1)_Sia_(1)_ glycan mediated ITGB1-RAP1B interaction (left). Hex_(5)_HexNAc_(4)_Fuc_(1)_Sia_(2)_ glycan mediated ITGB1-RAP1B interaction (right). Protein complexes from the literature and GPX results; interaction space models were calculated using DisVis, and docking models were generated using HADDOCK.

Further utility of this method was to further probe the role of glycans in protein complexes on the cell membrane. Based on the GPX data, we identified protein complexes that are known to interact from previous literature and can now be characterized by their glycan–protein interactions. We used the GPX results to map known protein–protein interactions on the cell membrane while inserting glycans into their respective interactions. For example, the Hex_(5)_HexNAc_(4)_Fuc_(1)_Sia_(1)_ and Hex_(5)_HexNAc_(4)_Fuc_(1)_Sia_(2)_ glycans on ITGB1 Asn97 were found to cross-link with the RAP1B protein at Lys104. ITGA5 (integrin alpha-5) has been previously shown to interact with ITGB1 (integrin beta-1) using X-ray crystallography.^32^ From the STRING database, RAP1B was predicted to interact with ITGB1 although the nature of this interaction is not well defined. We employed DisVis to corroborate the cross-linking results using distance restraints between the glycosylation site (Asn97) and the cross-linking site (RAP1B-Lys104) calculated with specific glycan lengths (10–30 Å).^33^ Putative ITGAV-ITGB1-RAP1B complexes were then generated using the HADDOCK software (Fig. S8†).^34^ Out of 66 structures, the complex with the highest HADDOCK score was subsequently glycosylated using the CHARMM-GUI glycan modeler generating two complexes each glycosylated with Hex_(5)_HexNAc_(4)_Fuc_(1)_Sia_(1)_ and Hex_(5)_HexNAc_(4)_Fuc_(1)_Sia_(2)_ (Fig. 4a). To show how the ITGB1-Asn97 glycans (Hex_(5)_HexNAc_(4)_Fuc_(1)_Sia_(1)_ and Hex_(5)_HexNAc_(4)_Fuc_(1)_Sia_(2)_) may interact with RAP1B, we mapped the residue interactions in Chimera after applying contact parameters (VDW overlap of −0.4 Å).^35^ The interaction model of RAP1B to the integrin complex suggested that the RAP1B molecule was rich in clusters of charged residues (Lys and Arg) that attracted sialic acids on the ITGB1, which can increase their binding affinities. The global structural similarity was quantified by using root-mean-square deviation (RMSD), and we observed that the ITGAV-ITGB1-RAP1B complex does not show significant conformational changes between the two glycoforms Hex_(5)_HexNAc_(4)_Fuc_(1)_Sia_(1)_ and Hex_(5)_HexNAc_(4)_Fuc_(1)_Sia_(2)_ (Cα RMSD = 0.004 Å, 1195 atoms). This observation was consistent with the previous molecular dynamics studies where the minimal differences in protein conformation and dynamics between glycosylated and deglycosylated proteins were noted.^34^ Meanwhile, we observed differences in residue contacts between Hex_(5)_HexNAc_(4)_Fuc_(1)_Sia_(1)_ and Hex_(5)_HexNAc_(4)_Fuc_(1)_Sia_(2)_, with the latter having fewer interactions (Fig. 4b) potentially contributing to the difference in biological activity. Overall, these GPX results offered unprecedented views of the interactions between proteins that are mediated by specific glycan types on cell membranes.

Finally, we identified a potential functional relevance of cell surface sialic acid-mediated protein networks. While it is not yet possible to interrupt a single glycoprotein–protein interaction, we may examine the general systemic effect by disrupting the sialic acid network and performing a standard cellular assay such as cell migration. We treated PNT2 cells with 3-fluorinated sialic acid, a sialyltransferase inhibitor, to reduce sialylation on the cell surface. As a result, PNT2 cell migration was significantly increased after the treatment (Fig. S9†), which demonstrated that the loss of sialic acids enhanced the growth of cells to make them more tumorigenic. Indeed, we previously found that cancerous prostate cells (LNCaP cells) have limited sialic acid expression compared to the normal prostate cells (PNT2 cells).^36^

## 4. Discussion

The cross-linking method coupled with cell membrane enrichment procedures reveals the highly interactive environment of glycan–peptide interactions of protein complexes and protein networks in the glycocalyx of cells. Cross-linking methods involving peptide–peptide interactions using specifically LC-MS methods and proteomic workflows have been well established.^37–39^ The addition of glycan–peptide interactions to these methods will further confirm peptide–peptide cross-linking results as well as establish the role of the glycans in these interactions. However, several technical limitations had made this concept previously impractical. These limitations were addressed to provide the first comprehensive characterization of glycan mediated protein–protein interactions *in situ*. The first major limitation was the lack of specific reactivity of the glycan toward a cross linker. Others have used chemical methods such as the oxidation of glycans with periodate, to produce a reactive aldehyde to react with reagents such as aminooxy or hydrazide moieties.^40^ However, due to the harsh oxidative conditions of this reaction, transient and weak interactions on the cell surfaces were lost or altered. The metabolic glycan modification used in this study is robust and highly effective. While it currently limits the interactions to those mediated by sialic acids, other activated monosaccharides such as fucose can now be developed.

A further limitation was the lack of available software to identify GPX products in the LC-MS/MS data. The fragmentation patterns of GPX products are more complicated and require knowledge of glycan fragmentation. A protein–protein cross-linking software tool (MeroX) was adapted by treating the whole glycan plus linker as a single component in a peptide–peptide cross link. Each glycan composition was therefore a different cross-linker and was searched individually. For example, a bi-antennary monosialylated glycan (Hex_(5)_HexNAc_(4)_Sia_(1)_) with composition and associated mass was deemed a different cross-linker from the bi-antennary glycan with a fucose (Hex_(5)_HexNAc_(4)_Fuc_(1)_Sia_(1)_). We generally examined the most abundant SiaNAzylated glycoforms expressed on the PNT2 cell membrane. This corresponded to nine glycoforms that were individually annotated using the MeroX software.

The proteins identified by GPXs were all mediated by sialic acids. Thus, the majority of the target proteins were likely sialic-acid binding proteins. Indeed, a comparison of the target proteins in this method and sialic acid binding proteins identified on the cell membrane of the same cell line by proximity oxidative labeling showed at least a 50% overlap between the two methods.^12^ However, although sialic acids were the basis of the cross-linking reaction, the interactions could have been driven by other glycan features present as part of the total structure. Thus, a terminal galactose or terminal fucose could have been the mediator of the glycan–peptide interaction while the sialic acid was merely an observer that produced the cross-link. The GPX between proteins, mediated by the glycans, could therefore be potentially structurally broader involving other types of (non-sialylated) glycan–peptide interactions. On the other hand, interactions among glycans with no sialic acid were not represented here. However, cross-linking of activated residues such as those based on fucose and *N*-acetylglucosamines could be performed in the future using the methods developed for sialic acid.

GPX has revealed the potential roles of specific glycoforms and provided additional clues regarding the large heterogeneity often associated with glycan structures. For example, fucose is a key monosaccharide residue involved in many biological processes, most notably recognition. However, the role of multiple fucosylation ranging from zero to greater than four on an *N*-glycan is a mystery. We can at least understand the difference between no fucose and one. The first fucose is generally found as a (1,6)-link on the chitobiose core. The complete loss of core fucosylation is generally lethal to humans.^41^ We found that adding a core fucose increased *trans* protein interactions. Thus, core fucosylation is potentially necessary to increase *trans* protein interaction, while no fucose can limit these interactions to favor *cis*-protein interactions. Further analysis of other proteins and other cell lines may similarly reveal the role of multiple fucosylation and multiple sialylation.

Sialic acids themselves are similarly important monosaccharides and are often found in cell membranes. The loss of sialic acid is not lethal as some cell lines are devoid of sialic acid in their *N*-glycans; however, altering the overall amount of sialic acids or sialylated glycans have been correlated with diseases such as cancer.^42^ Indeed, a decrease in sialic acid glycosylation has been associated with cancer progression. In the cell lines studied here, suppressing sialylation of glycans increased the tumorigenic potential of the cell. Increasing the number of sialic acids likely increases the strength of the glycan–peptide interaction. The most striking feature of the GPX results is the highly interactive networks of membrane proteins mediated by sialic acids and the rise of specific protein hubs. The outward hubs are glycoproteins that simultaneously interact with a large number of proteins through their glycans. Thus, integrin beta-1 (ITB1) and its partner integrin subunit alpha 3 are major hub proteins providing glycans that interact with the largest number of proteins (49 and 42 distinct proteins, respectively). As these proteins play important roles as receptors and in signaling, their high interactivity is consistent with these roles. However, proteins such as AMPN, which are known primarily to aid in the digestion of peptides from proteins as part of the gastric and pancreatic processes appear to similarly be highly interactive. AMPN may be an important protein with other functions that are yet to be revealed. Alternatively, the GPX may simply identify the peptides that were cross-linked in the process of being digested. Nonetheless, these interactive maps may eventually reveal the roles of the hubs in cells and the central roles they play in cellular functions.

## 5. Conclusion

Glycan–protein cross-linking adds a new dimension to protein–protein interactions. Traditional protein cross-linking methods reveal polypeptide–polypeptide interactions. GPX can identify glycan-mediated interactions that further localize the contact between proteins. This fills an important gap at a fortuitous period when glycomic methods are advancing significantly, while the essential contributions of glycosylation in physiological events such as immune regulation and cancer development are being explored. Glycan–protein cross-linking provides vast information that can be discovered, and it opens the door towards an exploration of pivotal functions of glycans in mediating fundamental cellular and molecular processes in native physiological conditions. The glycocalyx is indeed a highly interactive environment that can now be characterized with regard to the extent and nature of the interaction through glycan–protein cross-linking methods.

## Data availability

Raw mass spectrometry data are freely available and can be found on the MassIVE repository (DOI: 10.25345/C5VV5S, MSV000087442).

## Author contributions

Y. X., S. C., Q. L., Y. S., and G. X. performed the experiments. Y. X., S. C., Q. L., G. X., and C. B. L. designed the study. Y. X., S. C., M. R. A., J. R., and K. S. analyzed data. Y. X., S. C., and C. B. L. wrote the manuscript. C. B. L. supervised the study. All authors reviewed, edited and approved the manuscript prior to submission.

## Conflicts of interest

There are no conflicts with this report.

## Supplementary Material

SC-012-D1SC00814E-s001

SC-012-D1SC00814E-s002

## References

[cit1] Marth J. D., Grewal P. K. (2008). Mammalian glycosylation in immunity. Nat. Rev. Immunol..

[cit2] Kailemia M. J., Park D., Lebrilla C. B. (2017). Glycans and glycoproteins as specific biomarkers for cancer. Anal. Bioanal. Chem..

[cit3] Ruhaak L. R., Xu G., Li Q., Goonatilleke E., Lebrilla C. B. (2018). Mass Spectrometry Approaches to Glycomic and Glycoproteomic Analyses. Chem. Rev..

[cit4] Liu X. R., Zhang M. M., Gross M. L. (2020). Mass Spectrometry-Based Protein Footprinting for Higher-Order Structure Analysis: Fundamentals and Applications. Chem. Rev..

[cit5] Yu C., Huang L. (2018). Cross-Linking Mass Spectrometry: An Emerging Technology for Interactomics and Structural Biology. Anal. Chem..

[cit6] Huttlin E. L., Ting L., Bruckner R. J., Gebreab F., Gygi M. P., Szpyt J., Tam S., Zarraga G., Colby G., Baltier K., Dong R., Guarani V., Vaites L. P., Ordureau A., Rad R., Erickson B. K., Wühr M., Chick J., Zhai B., Kolippakkam D., Mintseris J., Obar R. A., Harris T., Artavanis-Tsakonas S., Sowa M. E., De Camilli P., Paulo J. A., Harper J. W., Gygi S. P. (2015). The BioPlex Network: A Systematic Exploration of the Human Interactome. Cell.

[cit7] Zhang M. M., Huang R. Y. C., Beno B. R., Deyanova E. G., Li J., Chen G., Gross M. L. (2020). Epitope and Paratope Mapping of PD-1/Nivolumab by Mass Spectrometry-Based Hydrogen–Deuterium Exchange, Cross-linking, and Molecular Docking. Anal. Chem..

[cit8] Cohen M. (2015). Notable Aspects of Glycan-Protein Interactions. Biomolecules.

[cit9] Tanaka Y., Kohler J. J. (2008). Photoactivatable Crosslinking Sugars for Capturing Glycoprotein Interactions. J. Am. Chem. Soc..

[cit10] Ramya T. N. C., Weerapana E., Liao L., Zeng Y., Tateno H., Liao L., Yates J. R., Cravatt B. F., Paulson J. C. (2010). In Situ trans Ligands of CD22 Identified by Glycan-Protein Photocross-linking-enabled Proteomics. Mol. Cell. Proteomics.

[cit11] Han S., Collins B. E., Bengtson P., Paulson J. C. (2005). Homomultimeric complexes of CD22 in B cells revealed by protein-glycan cross-linking. Nat. Chem. Biol..

[cit12] Li Q., Xie Y., Xu G., Lebrilla C. B. (2019). Identification of potential sialic acid binding proteins on cell membranes by proximity chemical labeling. Chem. Sci..

[cit13] Frei A. P., Jeon O.-Y., Kilcher S., Moest H., Henning L. M., Jost C., Plückthun A., Mercer J., Aebersold R., Carreira E. M., Wollscheid B. (2012). Direct identification of ligand-receptor interactions on living cells and tissues. Nat. Biotechnol..

[cit14] Sobotzki N., Schafroth M. A., Rudnicka A., Koetemann A., Marty F., Goetze S., Yamauchi Y., Carreira E. M., Wollscheid B. (2018). HATRIC-based identification of receptors for orphan ligands. Nat. Commun..

[cit15] Li Q., Xie Y., Wong M., Barboza M., Lebrilla C. B. (2020). Comprehensive structural glycomic characterization of the glycocalyxes of cells and tissues. Nat. Protoc..

[cit16] Iacobucci C., Götze M., Ihling C. H., Piotrowski C., Arlt C., Schäfer M., Hage C., Schmidt R., Sinz A. (2018). A cross-linking/mass spectrometry workflow based on MS-cleavable cross-linkers and the MeroX software for studying protein structures and protein–protein interactions. Nat. Protoc..

[cit17] Mädler S., Bich C., Touboul D., Zenobi R. (2009). Chemical cross-linking with NHS esters: a systematic study on amino acid reactivities. J. Mass Spectrom..

[cit18] Chen Z.-L., Meng J.-M., Cao Y., Yin J.-L., Fang R.-Q., Fan S.-B., Liu C., Zeng W.-F., Ding Y.-H., Tan D., Wu L., Zhou W.-J., Chi H., Sun R.-X., Dong M.-Q., He S.-M. (2019). A high-speed search engine pLink 2 with systematic evaluation for proteome-scale identification of cross-linked peptides. Nat. Commun..

[cit19] Liu F., Lössl P., Scheltema R., Viner R., Heck A. J. R. (2017). Optimized fragmentation schemes and data analysis strategies for proteome-wide cross-link identification. Nat. Commun..

[cit20] Kechagia J. Z., Ivaska J., Roca-Cusachs P. (2019). Integrins as biomechanical sensors of the microenvironment. Nat. Rev. Mol. Cell Biol..

[cit21] Masedunskas A., King J. A., Tan F., Cochran R., Stevens T., Sviridov D., Ofori-Acquah S. F. (2006). Activated leukocyte cell adhesion molecule is a component of the endothelial junction involved in transendothelial monocyte migration. FEBS Lett..

[cit22] Sarafian V., Jadot M., Foidart J.-M., Letesson J.-J., Van den Brûle F., Castronovo V., Wattiaux R., Wattiaux-De Coninck S. (1998). Expression of Lamp-1 and Lamp-2 and their interactions with galectin-3 in human tumor cells. Int. J. Cancer.

[cit23] Li J., Deffieu M. S., Lee P. L., Saha P., Pfeffer S. R. (2015). Glycosylation inhibition reduces cholesterol accumulation in NPC1 protein-deficient cells. Proc. Natl. Acad. Sci. U. S. A..

[cit24] Davis T. A., Loos B., Engelbrecht A. M. (2014). AHNAK: The giant jack of all trades. Cell. Signalling.

[cit25] Chen B., Wang J., Dai D., Zhou Q., Guo X., Tian Z., Huang X., Yang L., Tang H., Xie X. (2017). AHNAK suppresses tumour proliferation and invasion by targeting multiple pathways in triple-negative breast cancer. J. Exp. Clin. Cancer Res..

[cit26] Shaikh F. M., Seales E. C., Clem W. C., Hennessy K. M., Zhuo Y., Bellis S. L. (2008). Tumor cell migration and invasion are regulated by expression of variant integrin glycoforms. Exp. Cell Res..

[cit27] Mohamed A., Shah A. D., Chen D., Hill M. M. (2019). RaftProt V2: understanding membrane microdomain function through lipid raft proteomes. Nucleic Acids Res..

[cit28] Takahashi M., Kuroki Y., Ohtsubo K., Taniguchi N. (2009). Core fucose and bisecting GlcNAc, the direct modifiers of the N-glycan core: their functions and target proteins. Carbohydr. Res..

[cit29] Otto V. I., Schürpf T., Folkers G., Cummings R. D. (2004). Sialylated Complex-type N-Glycans Enhance the Signaling Activity of Soluble Intercellular Adhesion Molecule-1 in Mouse Astrocytes. J. Biol. Chem..

[cit30] Yen H.-Y., Liu Y.-C., Chen N.-Y., Tsai C.-F., Wang Y.-T., Chen Y.-J., Hsu T.-L., Yang P.-C., Wong C.-H. (2015). Effect of sialylation on EGFR phosphorylation and resistance to tyrosine kinase inhibition. Proc. Natl. Acad. Sci. U. S. A..

[cit31] Murrey H. E., Ficarro S. B., Krishnamurthy C., Domino S. E., Peters E. C., Hsieh-Wilson L. C. (2009). Identification of the Plasticity-Relevant Fucose-α(1−2)-Galactose Proteome from the Mouse Olfactory Bulb. Biochemistry.

[cit32] Nagae M., Re S., Mihara E., Nogi T., Sugita Y., Takagi J. (2012). Crystal structure of α5β1 integrin ectodomain: Atomic details of the fibronectin receptor. J. Cell Biol..

[cit33] van Zundert G. C. P., Bonvin A. M. J. J. (2015). DisVis: quantifying and visualizing accessible interaction space of distance-restrained biomolecular complexes. Bioinformatics.

[cit34] van Zundert G. C. P., Rodrigues J. P. G. L. M., Trellet M., Schmitz C., Kastritis P. L., Karaca E., Melquiond A. S. J., van Dijk M., de Vries S. J., Bonvin A. M. J. J. (2016). The HADDOCK2.2 Web Server: User-Friendly Integrative Modeling of Biomolecular Complexes. J. Mol. Biol..

[cit35] Pettersen E. F., Goddard T. D., Huang C. C., Couch G. S., Greenblatt D. M., Meng E. C., Ferrin T. E. (2004). UCSF Chimera—A visualization system for exploratory research and analysis. J. Comput. Chem..

[cit36] Xie Y., Sheng Y., Li Q., Ju S., Reyes J., Lebrilla C. B. (2020). Determination of the glycoprotein specificity of lectins on cell membranes through oxidative proteomics. Chem. Sci..

[cit37] Mintseris J., Gygi S. P. (2020). High-density chemical cross-linking for modeling protein interactions. Proc. Natl. Acad. Sci. U. S. A..

[cit38] Götze M., Iacobucci C., Ihling C. H., Sinz A. (2019). A Simple Cross-Linking/Mass Spectrometry Workflow for Studying System-wide Protein Interactions. Anal. Chem..

[cit39] Chavez J. D., Lee C. F., Caudal A., Keller A., Tian R., Bruce J. E. (2018). Chemical Crosslinking Mass Spectrometry Analysis of Protein Conformations and Supercomplexes in Heart Tissue. Cell Syst..

[cit40] Rouhanifard S. H., Nordstrøm L. U., Zheng T., Wu P. (2013). Chemical probing of glycans in cells and organisms. Chem. Soc. Rev..

[cit41] Schneider M., Al-Shareffi E., Haltiwanger R. S. (2017). Biological functions of fucose in mammals. Glycobiology.

[cit42] Varki A. (2008). Sialic acids in human health and disease. Trends Mol. Med..

