# Supplementary material for: Glycan–protein cross-linking mass spectrometry reveals sialic acid-mediated protein networks on cell surfaces
Source: Chem Sci. 2021 May 18;12(25):8767–77. doi: 10.1039/d1sc00814e (PMC8246274; doi:10.1039/d1sc00814e)
Supplement: SC-012-D1SC00814E-s002 [file SC-012-D1SC00814E-s002.pdf]

Electronic Supplementary Material (ESI) for Chemical Science.
This journal is © The Royal Society of Chemistry 2021

Glycan-protein cross-linking mass spectrometry reveals sialic acid-mediated protein networks on cell surfaces

Authors

Yixuan Xie[1]; Siyu Chen[1]; Qiongyu Li[1]; Ying Sheng[2], Michael Russelle Alvarez[4], Joe Reyes[5], Gege Xu[1], Kemal Solakyildirim,[1,6] Carlito B. Lebrilla[1,3]

[1]Department of Chemistry, University of California, Davis, Davis, California, United States. [2]Department of Chemistry, Biochemistry, Molecular, Cellular and Developmental Biology Graduate Group, University of California, Davis, Davis, California, United States. [3]Department of Biochemistry, University of California, Davis, Davis, California, United States. [4]Institute of Chemistry, University of the Philippines Los Baños, Laguna, Philippines. [5]Marine Science Institute, University of the Philippines, Diliman, Quezon City, Philippines. [6]Department of Chemistry, Erzincan Binali Yildirim University, Erzincan, Turkey. Correspondence and requests for materials should be addressed to C.B.L. (email: cblebrilla@ucdavis.edu)

Synthesis of cross-linker NHS-cyclooctyne

We first synthesized the biocompatible cross-linker that contains cyclooctyne and NHS functional groups (**Figure S10**). Detailed procedures for the synthesis of the NHS-cyclooctyne cross-linker were described previously by *Bernardin et al*.[1] Briefly, 8,8-dibromobicyclo[5.0.1]octane was obtained by brominating cycloheptene with bromoform and was purified by filtration using hexane/EtOAc (95:5, v/v). (yield 80%). Methyl 2-bromocyclooct-1-en-3-glycolate was generated by adding methyl glycolate to the reaction product in dry toluene and silver perchlorate. The crude product was purified by silica gel chromatography (2-15% EtOAc in hexane, v/v). Subsequently, the product was added to a solution of sodium methanolate, and 2-(cyclooct-2-yne-1-yloxy)acetic acid was yielded produced as a slightly yellow oil. Purification was performed by silica gel chromatography (0-8% Methanol in DCM) (yield 60%). The yielded product 2-(cyclooct-2-yne-1-yloxy)acetic acid was dissolved in dry DCM (0.06 M) and further modified by stepwise addition of NHS (1.5 eq.) and EDC•HCl (1.5 eq). The solution was stirred overnight at room temperature under argon atmosphere. The final product was extracted from the organic layer by washing twice with H_2O and brine, and drying over $MgSO_4$. 2-(Ccyclooct-2-yne-1-yloxy)acetic acid-NHS-Ester was collected as slightly yellow oil by concentration under reduced pressure with a 70% yield. H-NMR (400 MHz; CDCl3): δ 4.56-4.38 (m, 2H), 3.68 (q, J = 13 6.1 Hz, 1H), 2.82 (s, 4H), 2.31-1.45 (m, 14H). The synthesis was readily scaled to produce larger amounts for our cross-linking studies. NMR was employed to characterize and confirm the structures (**Figure S11**).

Monitoring ManNAz incorporation using MS

The cross-linking reactions between glycans and proteins depended greatly on the efficiency of the ManNAz incorporation in the cell. To monitor the incorporation we used liquid chromatography-tandem mass spectrometry (LC-MS/MS) and profiled the resulting N-glycans using methods described previously.[2] The N-glycomic profile yielded over 300 sialylated N-glycans in the PNT2 cell line (**Figure S12**). The SiaNAz-modified glycans were readily identified based on their corresponding masses. Tandem MS was used to confirm the incorporation using diagnostic peaks corresponding to the SiaNAz cation (m/z = 333.10). To obtain maximum incorporation, we further monitored the expression of SiaNAz-containing N-glycans with regard to treatment time. As shown in **Figure S1a** and **b**, up to 90% of the total sialic acid could be converted to SiaNAz after 72 hours of ManNAz treatment.

Validation of cross-linking reaction on cell lines

To determine first the extent of the cross-linking reactions, we employed gel electrophoresis to analyze the products. The proteins were digested to peptides, and the molecular weight ranged from 3k Da to 5kDa with the cross-linker while the masses of around 2k Da were observed without the cross-linker modification (control) (**Figure S13a**).

Validation of enrichment method

With methods for analyzing the crosslink products, we optimized enrichment methods for the resulting GPX pairs. PPX products were commonly fractionated using strong cation exchange (SCX) or size exclusion chromatography (SEC), while glycopeptides were usually separated from peptide background using hydrophilic interaction chromatography (HILIC).[3,4] We tested the enrichment of GPX products using SCX cartridge and HILIC. As shown in **Figure S13b**, around 200 GPX pairs were identified using SCX, while only 30 GPX pairs were identified using the combination of SCX and HILIC. These results suggested that one-step SCX is sufficient for the enrichment of GPX products prior to MS analysis.

Validation of GPX analysis workflow

We further validated the workflow with lectins that are known to bind sialic acid. With the cross-linker in place, *Sambucus nigra* agglutinin (SNA) was reacted with the cell membrane containing SiaNAz (**Figure S14a**). From the LC-MS/MS data, the resulting GPX pairs included membrane glycopeptides with an SNA peptide. More than 100 of GPX products containing SNA peptides were identified, which corresponded to approximately 50 unique glycoprotein-SNA pairs. We compared the glycoproteins cross-linked by SNA to those previously identified as potentially SNA-binding proteins using a proximity approach (Lectin PROXL). In an earlier study, SNA was modified with Fe^{3+} probe to oxidize proteins that were in the proximity of SNA.[5] As shown in **Figure S14b**, a large fraction (60%) of SNA-cross-linked glycoproteins was also found by our previously developed method further validating the results of the cross-linking method.

Supplementary Figures

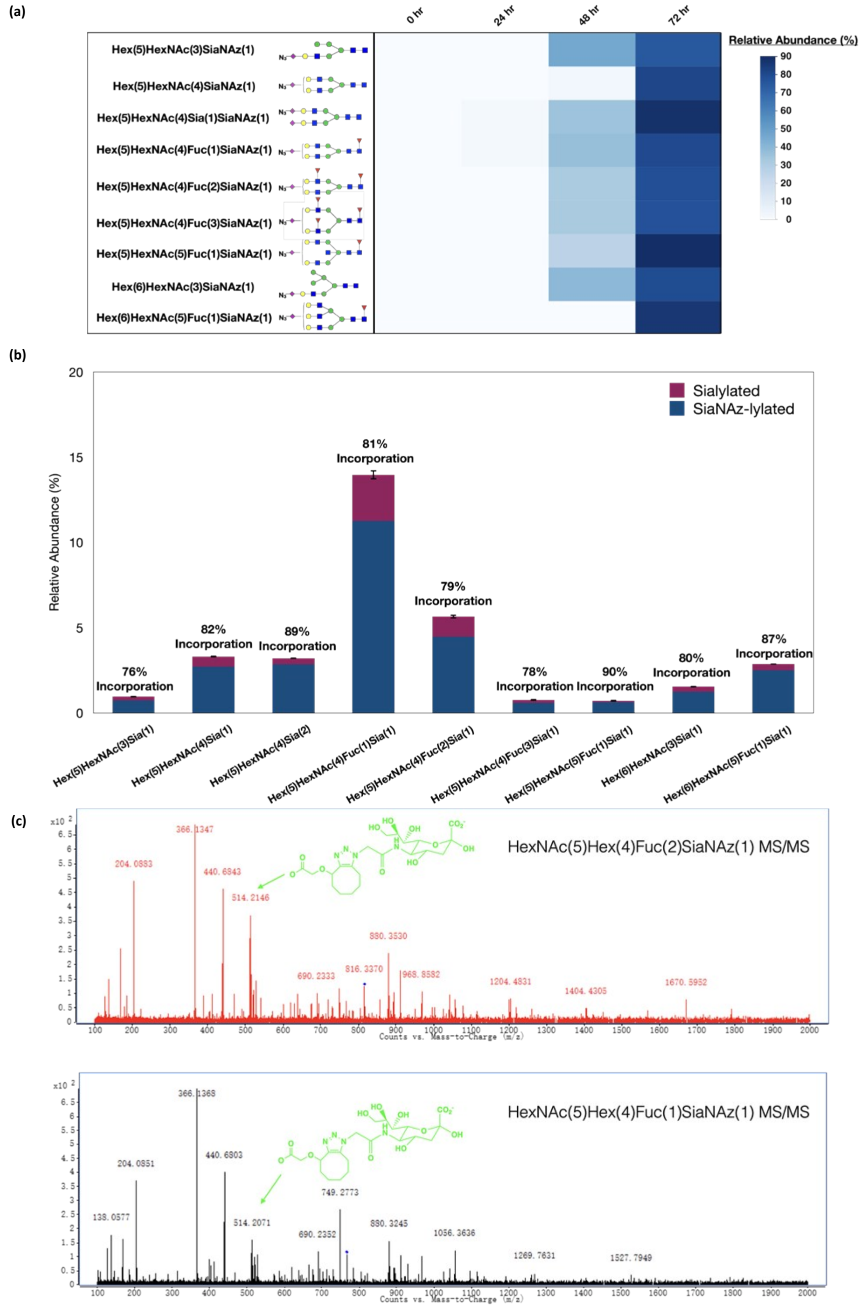

Figure S1 (**a**) The change of relative abundances of SiaNAzylated glycan subtypes over the time of the ManNAz treatment. (**b**) The relative abundances of the most abundant

SiaNAz-containing glycans in PNT2 cells. Different glycans have variable incorporation rate, while over 80% was achieved on average. (**c**) Tandem MS of released N-glycans from cell membrane after click addition of a crosslinker. The MS/MS spectra validate the composition and provide structural information regarding the reacted glycan.

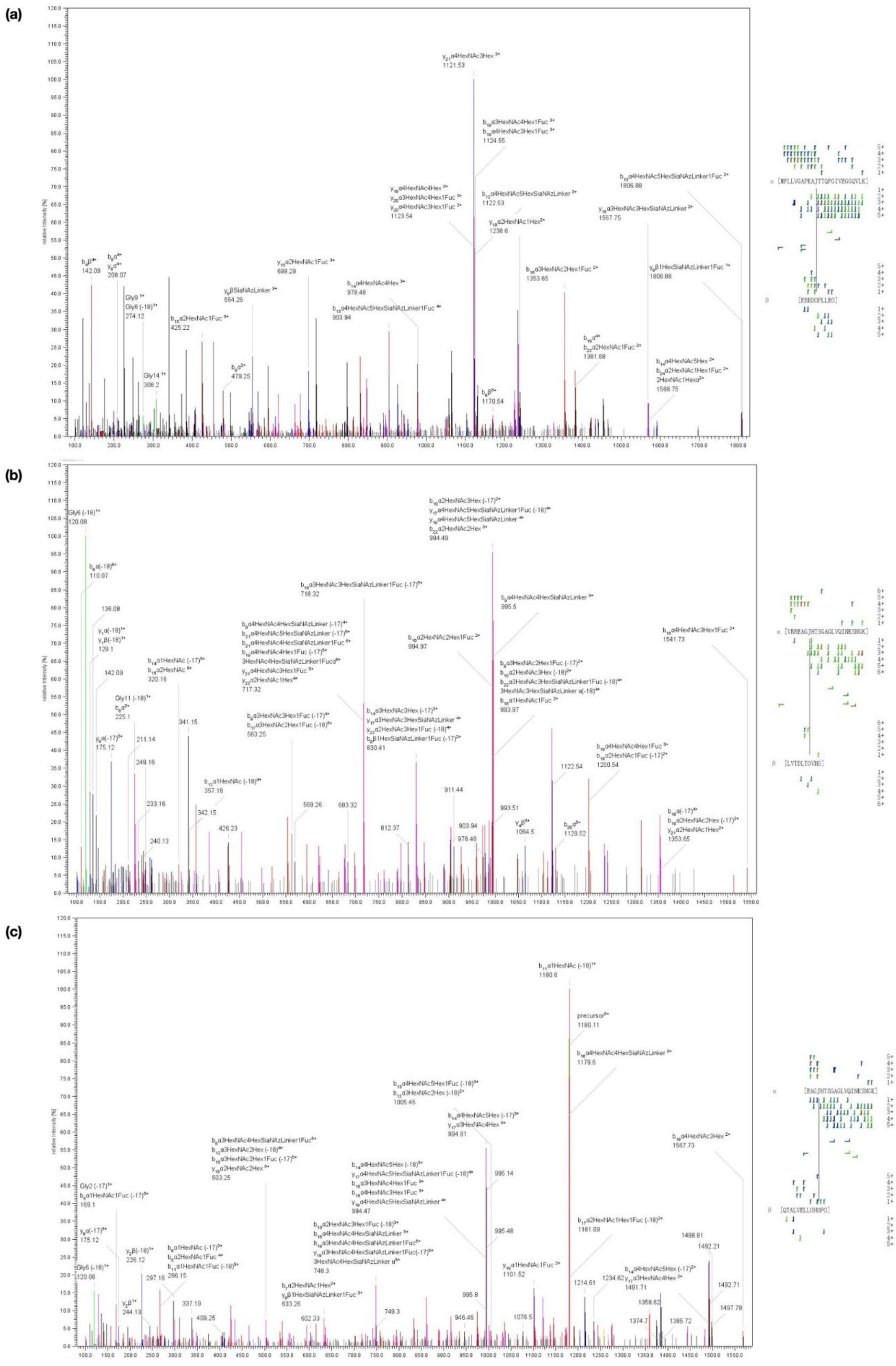
(a)
(b)
(c)

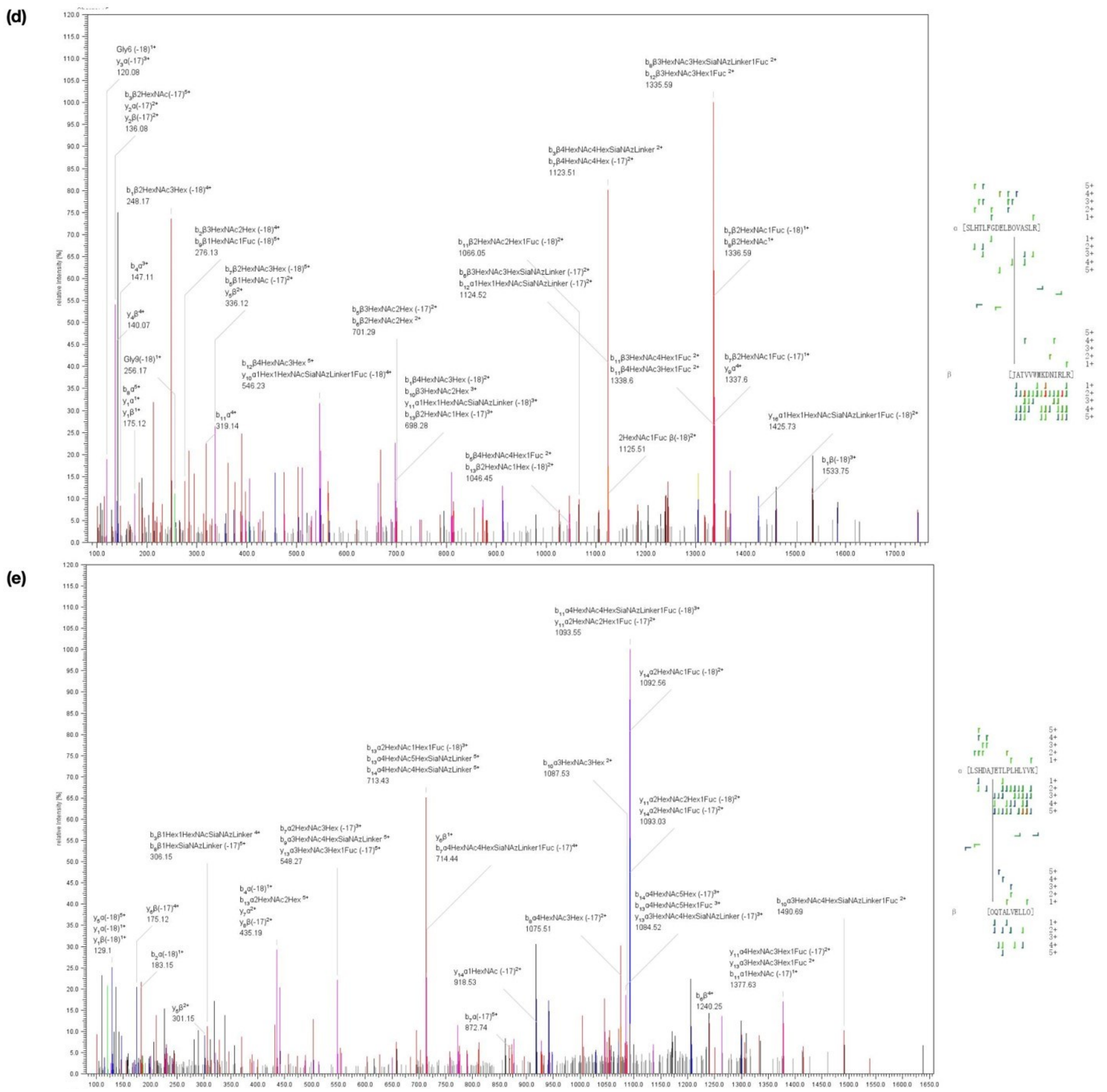

Figure S2 Representative identified tandem mass spectrums of cross-linked GPX pairs using MeroX software. (**a**) ALB(K304)-5411-ITAV(N74) (**b**) ALB(K263)-5421-MPRD(N83) (**c**) ALB(K559)-5421-MPRD(N83) (**d**) ALB(K100)-5431-L1CAM(N361) (**e**) ALB(K548)-5511-L1CAM(N733)

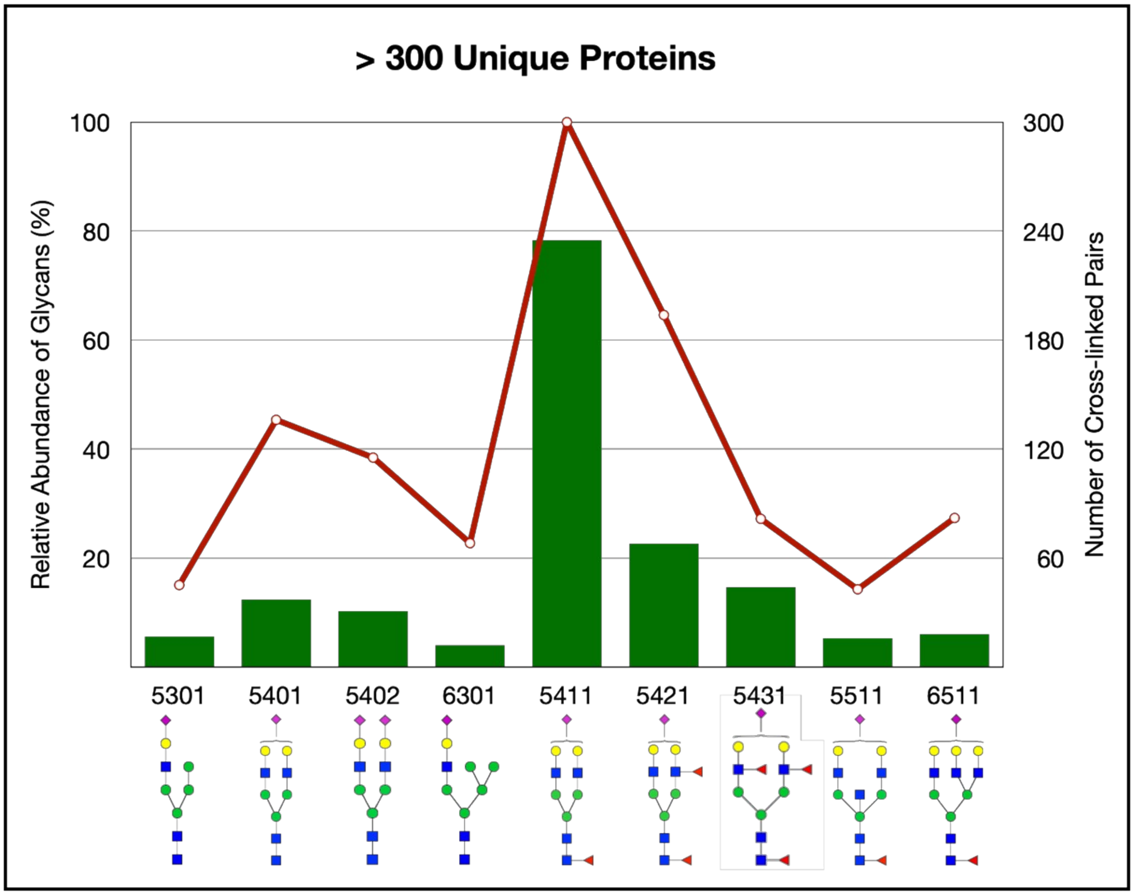

Figure S3 The number of GPX pairs associated with each glycoform. The monosialylated, monofucosylated biantennary glycan yields the largest number of GPX pairs.

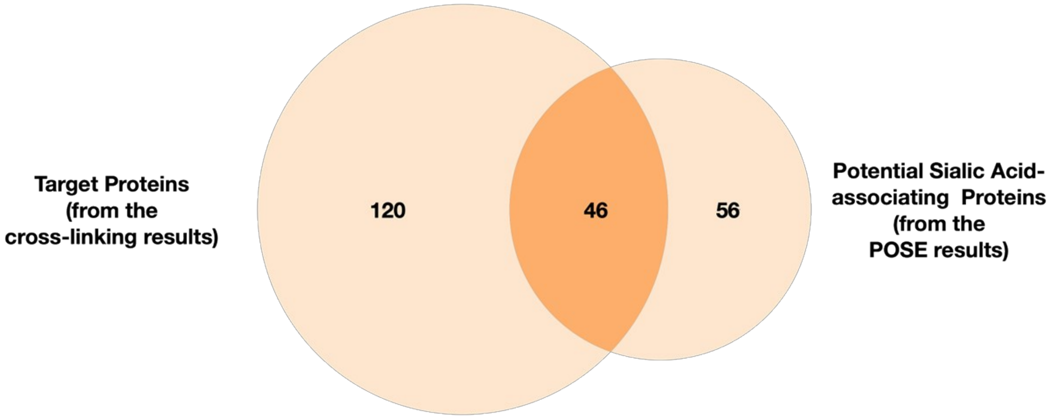

Figure S4 Target proteins captured by sialylated proteins were found to overlap with the sialic acid-associated proteins as determined by a previously developed method POSE.

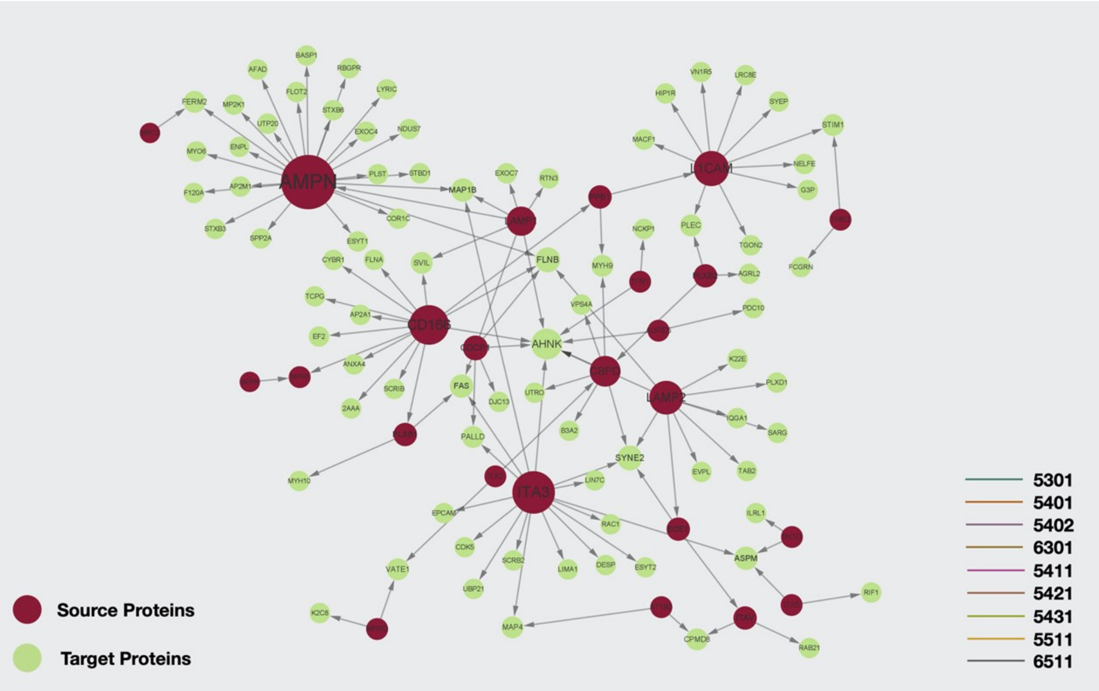

Figure S5 The unreviewed interactions between glycoproteins and proteins.

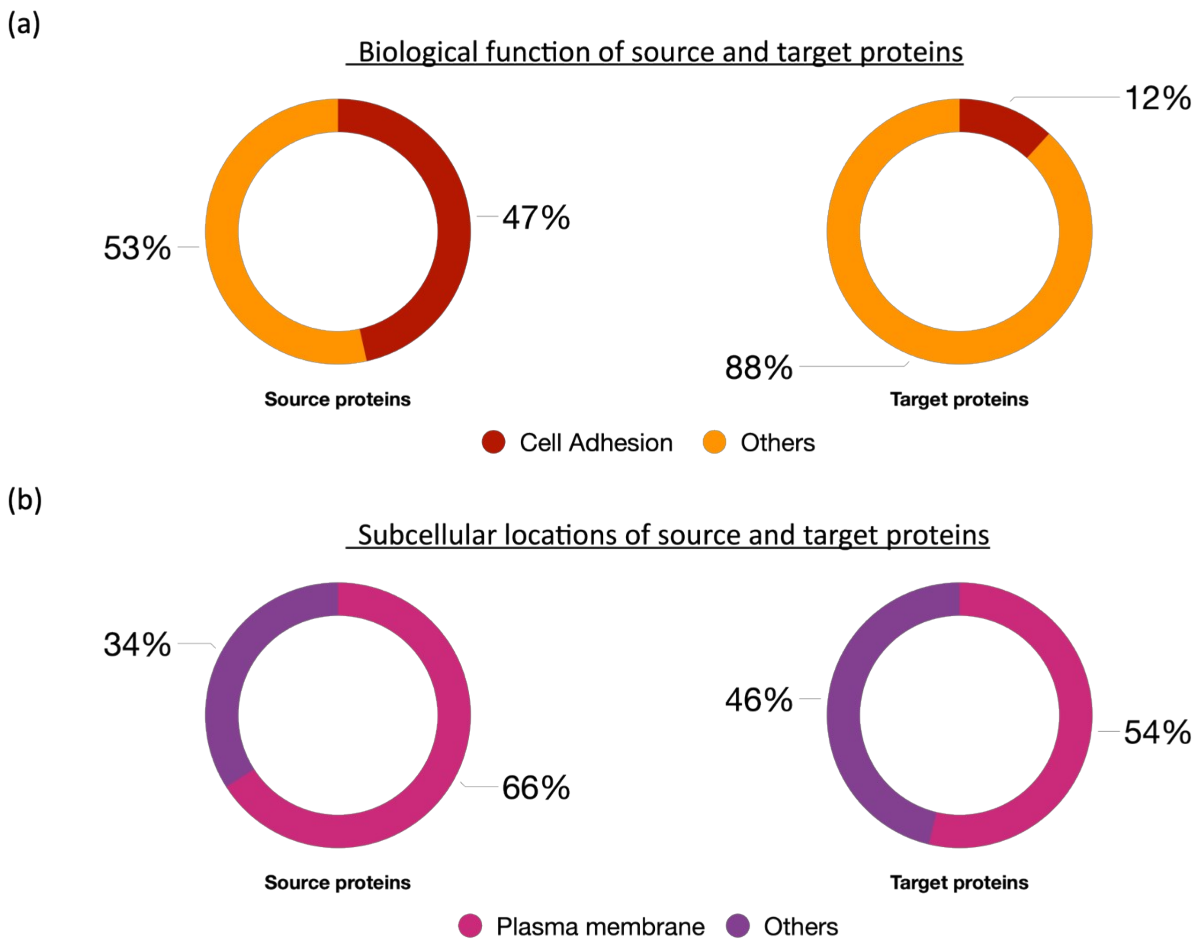

Figure S6 (**a**) The biological function of source (**left**) and target (**right**) proteins. (**b**) The subcellular locations of source (**left**) and target (**right**) proteins.

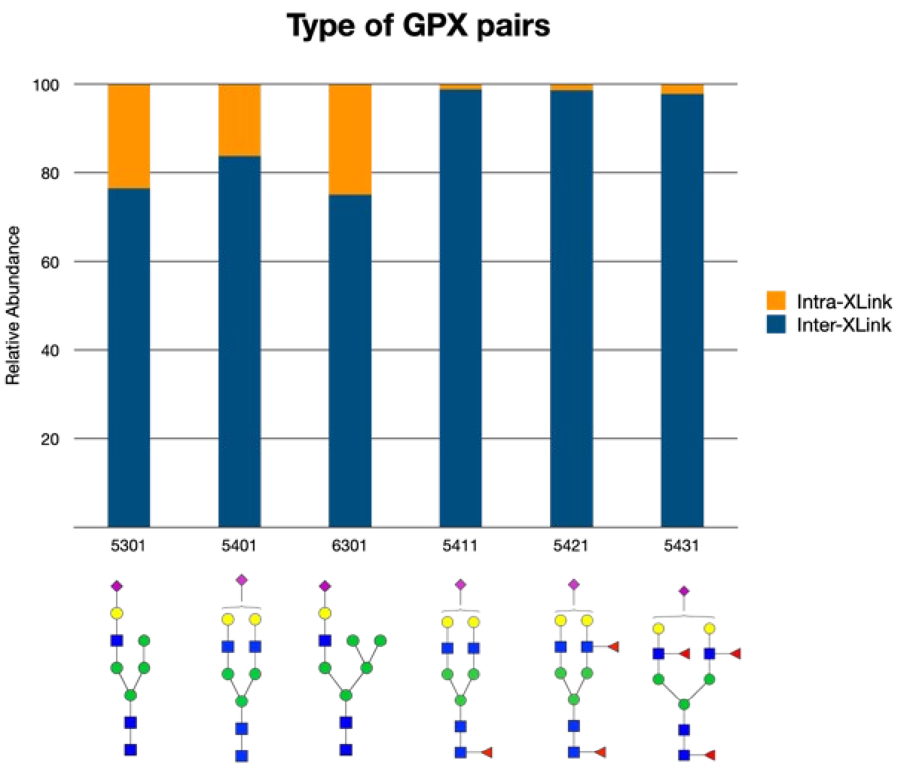

Figure S7 GPX analysis indicated that the glycan composition having core fucose interacted more with other proteins (inter) than itself (intra). Conversely, the absence of the fucose resulted in significantly more interact with the parent protein (intra).

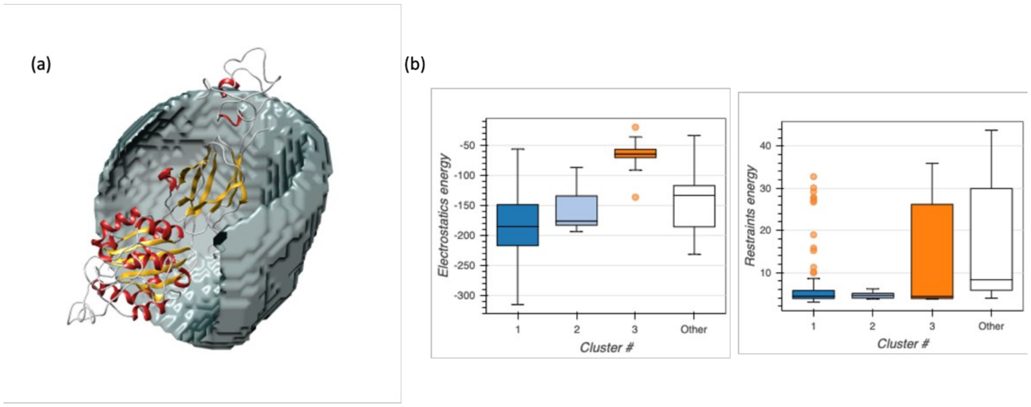

Figure S8 The accessible interaction space of distance-restrained RAP1B-ITGB1 complex analzyed using DisVis.

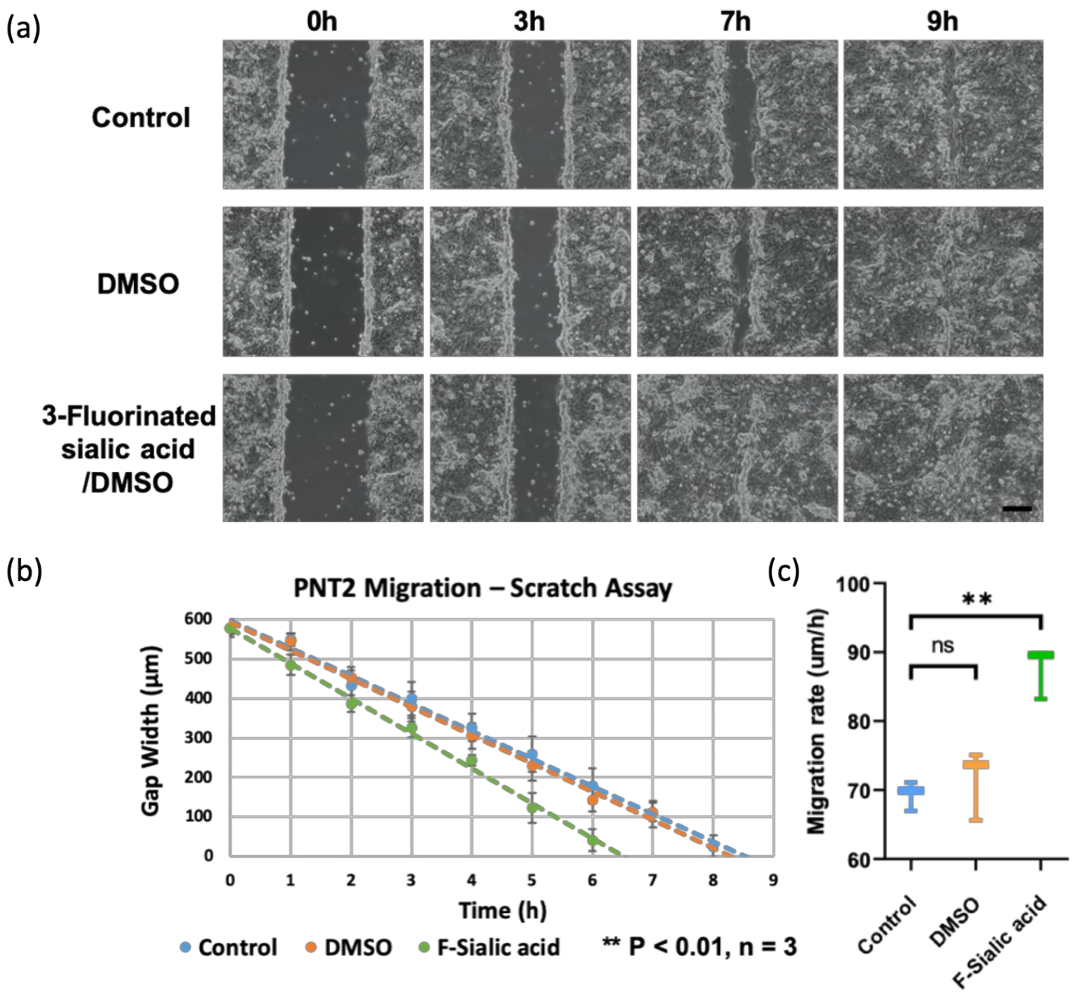

Figure S9 PNT2 migration was accelerated after cell surface sialic acid was lost. (**a**) Representative images show that treatment with the sialylation inhibitor, 3-fluorinated sialic acid, resulted in increased migration speed compared with controls. The black bar at the right lower corner is 200 µm. (**b**) Wound closure was expressed as the remaining gap width uncovered by the cells. The gap width at time 0 hour was normalized. (**c**) The significant difference in migration rate was observed between control (with sialic acid) and with sialic acid inhibitor treatment (without sialic acid).

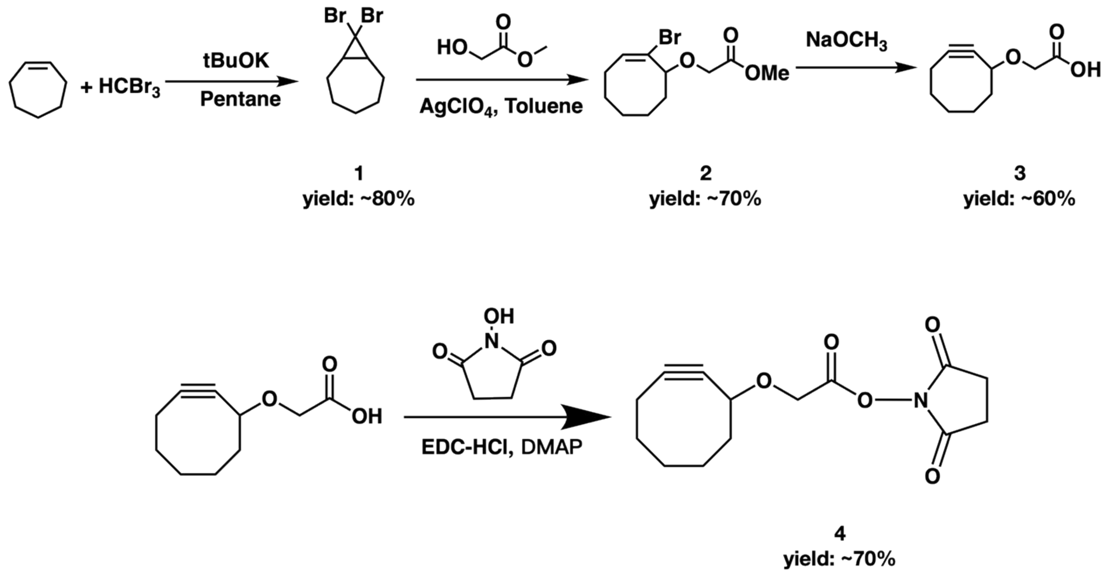

Figure S10 The synthetic route for the synthesis of the cross-linker, NHS-cyclooctyne. Around 40% total yield was achieved after four steps.

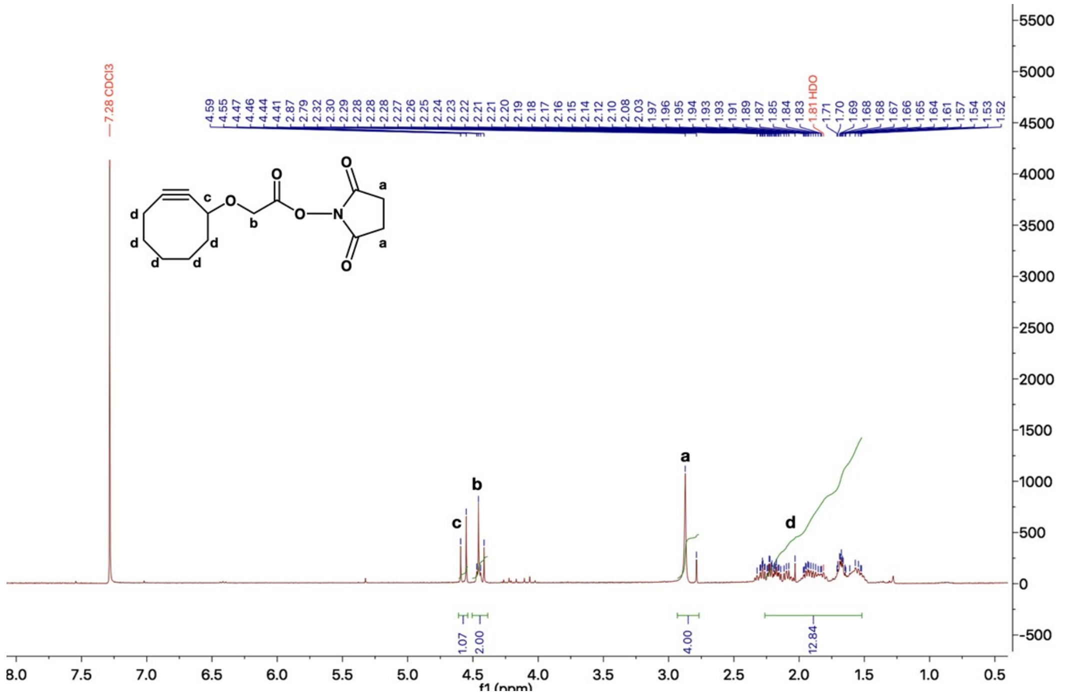

Figure S11 ^{1}H NMR spectra of NHS-cyclooctyne.

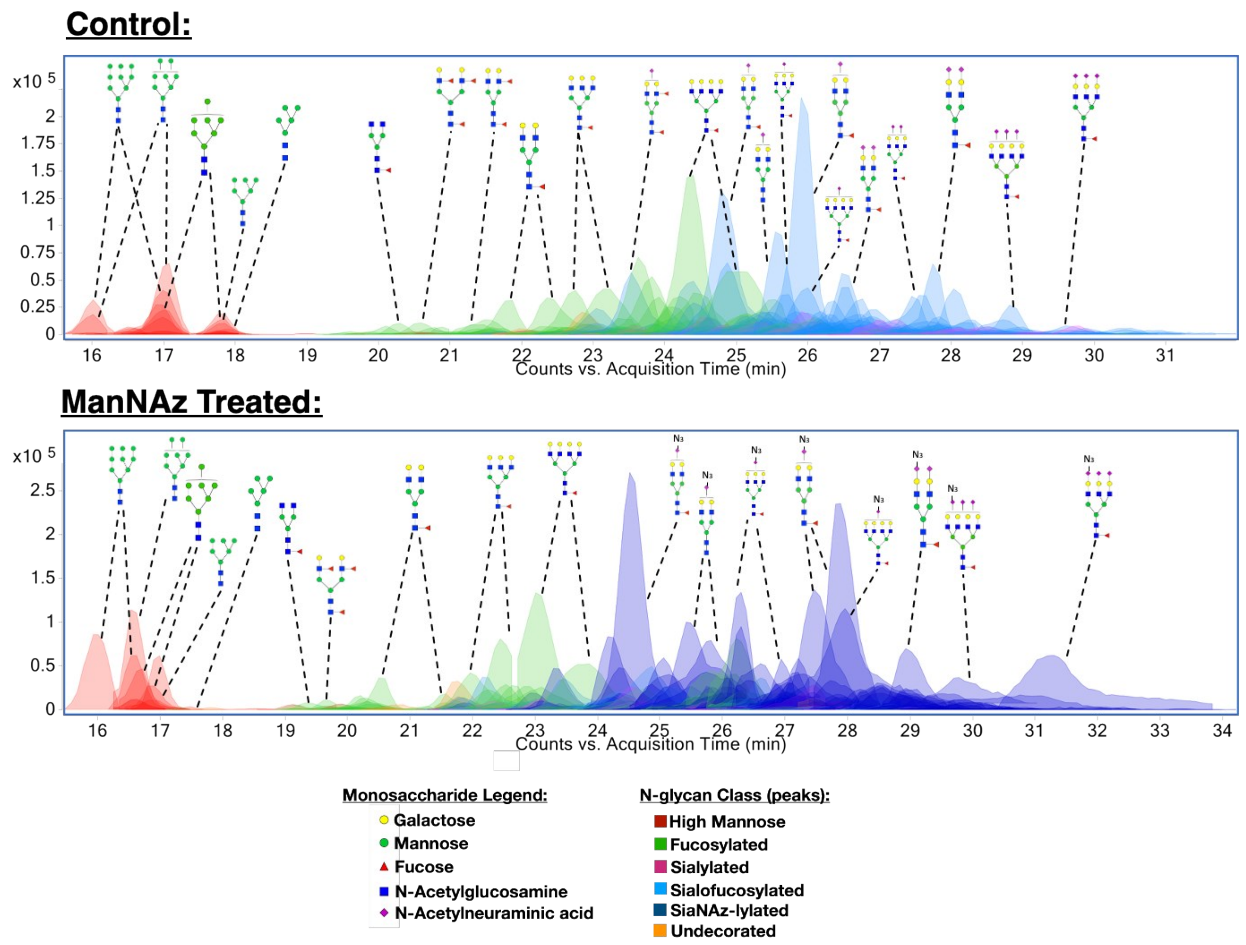

Figure S12 LC-MS profile of N-Glycans released from PNT2 cells (**top**). Annotated structures are putative based on mass and compositions. LC-MS peaks are color coded to assign glycan subtype. Over 300 structures are generally observed in a chromatogram. N-Glycan profile from cells after treatment with ManNAz (**bottom**). SiaNAz containing compounds are labeled accordingly.

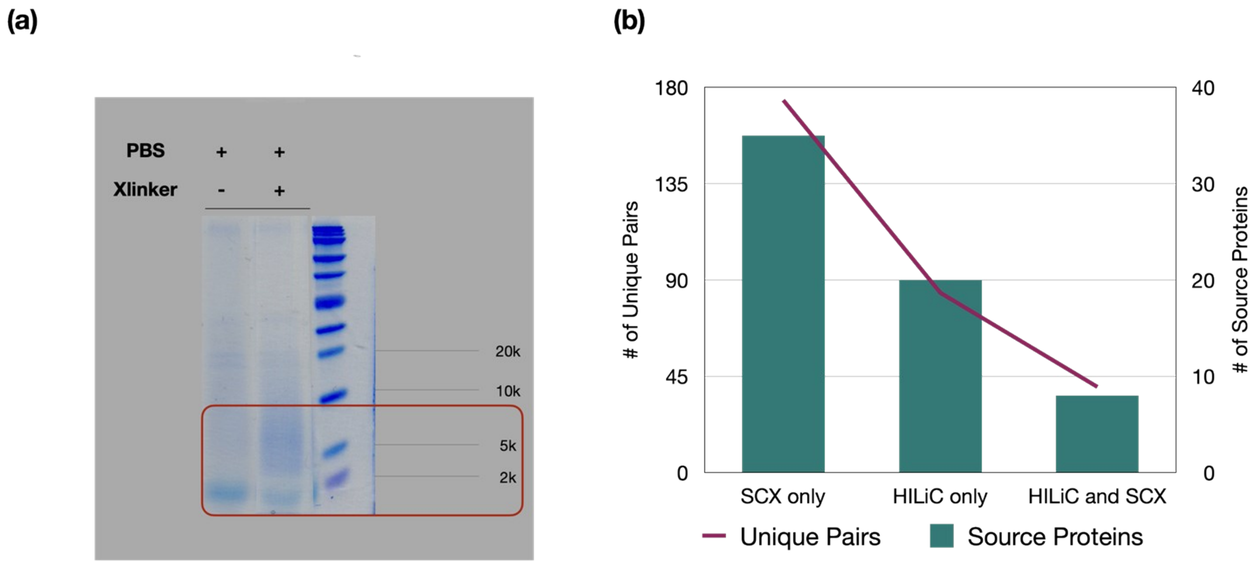

Figure S13 (**a**) SDS-PAGE results of digested samples from control (no cross-linker) and with the treatment of cross-linker. The molecular weight of most of the peptide was increased after the cross-linker treatment, which demonstrated the efficiency of the cross-linking reaction. (**b**) Performance of the different enrichment methods for GPX pairs.

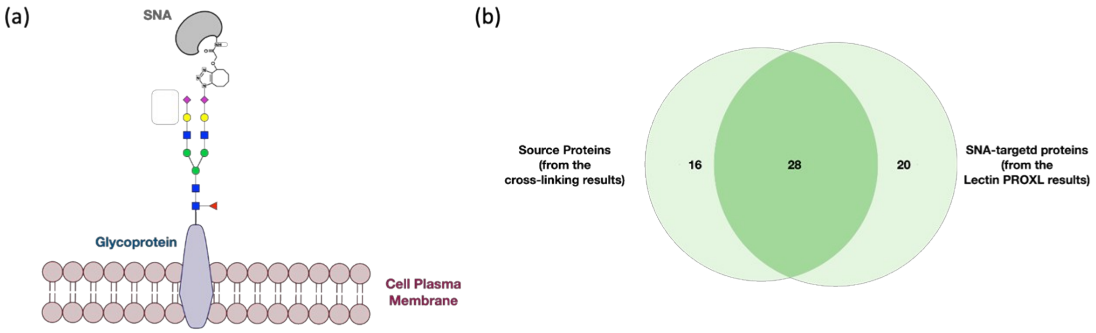

Figure S14 (**a**) Schematic diagram of the SNA-glycoprotein cross-linking on the cell membrane. (**b**) The overlap of SNA-labeled proteins between the cross-linking method and the proximity method.

References

1. Bernardin, A.; Cazet, A.; Guyon, L.; Delannoy, P.; Vinet, F.; Bonnaffé, D.; Texier, I., Copper-Free Click Chemistry for Highly Luminescent Quantum Dot Conjugates: Application to in Vivo Metabolic Imaging. *Bioconjugate Chemistry* **2010,** *21* (4), 583-588.
2. Park, D. D.; Xu, G.; Wong, M.; Phoomak, C.; Liu, M.; Haigh, N. E.; Wongkham, S.; Yang, P.; Maverakis, E.; Lebrilla, C. B., Membrane glycomics reveal heterogeneity and quantitative distribution of cell surface sialylation. *Chemical Science* **2018,** *9* (29), 6271-6285.
3. Li, Q.; Xie, Y.; Wong, M.; Lebrilla, C. B., Characterization of Cell Glycocalyx with Mass Spectrometry Methods. *Cells* **2019,** *8* (8).
4. Schmidt, R.; Sinz, A., Improved single-step enrichment methods of cross-linked products for protein structure analysis and protein interaction mapping. *Analytical and Bioanalytical Chemistry* **2017,** *409* (9), 2393-2400.
5. Xie, Y.; Sheng, Y.; Li, Q.; Ju, S.; Reyes, J.; Lebrilla, C. B., Determination of the glycoprotein specificity of lectins on cell membranes through oxidative proteomics. *Chemical Science* **2020**.
